# A Fully Distributed Protocol with an Event-Triggered Communication Strategy for Second-Order Multi-Agent Systems Consensus with Nonlinear Dynamics

**DOI:** 10.3390/s21124059

**Published:** 2021-06-12

**Authors:** Tao Li, Quan Qiu, Chunjiang Zhao

**Affiliations:** 1 Beijing Research Center of Intelligent Equipment for Agriculture, Beijing 100097, China; lit@nercita.org.cn; 2 National Engineering Research Center for Information Technology in Agriculture, Beijing 100097, China

**Keywords:** event-trigger, multi-agent systems, leader-following consensus, nonlinear dynamics

## Abstract

This paper presents the communication strategy for second-order multi-agent systems with nonlinear dynamics. To address the problem of the scarcity of communication channel resources and get rid of using continuous signals among the followers in lead-follower multi-agent systems, a novel event-triggered communication mechanism is proposed in this paper. To avoid employing the centralized information that depends on the Laplacian matrix spectrum, a network protocol with updated coupling gains is proposed, as well as an event-triggered strategy with updated thresholds. To eliminate the ill effects of inter-node communicating noise, relative positions are employed by the protocol instead of absolute positions. By a Lyapunov–Krasovskii functional, it is rigorously proven that the leader-following consensus of MASs is achieved without Zeno behavior, under the control of the proposed protocol with an event-triggered mechanism communication. The effectiveness of the proposed protocol is verified through numerical examples.

## 1. Introduction

Over the last decade, ever-increasing research trends concentrate on the studies of multi-agent systems (MASs). A fundamental problem of MASs is designing a networking protocol of consensus, which means that agents converge to a common point or state value. Consensus has been extensively investigated in the literature [1,2]. Reviewing the existing studies of this area, one may note that the effect of network-induced communicating constraints over the consensus control performance attracts extensive attention, such as the problems of communication delays [3], switching topology [4], and discrete inter-agent information exchange [5]. One of the main sources of communication constraints [6] is the scarce network bandwidth. Generally speaking, the communication channels of MASs are usually multipurpose and various kinds of inter-agent information share common channels. To achieve desired timeliness, with limited bandwidth, reducing the burden of communication is expected.

It is well-known that continuous signals among agents usually require more communication bandwidth than discrete signals. In light of this, the sample-based communicating mechanism is proposed to take samples of the exchange of information among agents. Under the control of a well-designed sample-based communicating mechanism, a consensus of MASs can be guaranteed with less channel occupancy. In the sample-based mechanisms, there are two kinds, including the event-triggering mechanism (ETM) and time-trigger mechanism (TTM). TTM takes the samples according to time, which is widely used in many existing sample-based network protocols [7,8]. However, one potential drawback is that the time-driven sampling, which is independent of system states, feedbacks, communication resources, etc., may result in unnecessary and redundant sampled-data [9,10]. To optimize the strategies of sampling, ETM is proposed, where the sampling is triggered by predefined conditions. The conditions depend on the system state, the feedback signals, or other artificially designed conditions.

ETM can be dated back to the late 1950s [11] and then the event-trigger mechanism has been extensively developed during the past decades in the control community. One of the pioneering works in the research of MAS consensus with ETM was presented by [12], where the control actuation is triggered whenever the errors become large enough with regard to the norm of the state. The proposed ETM guarantees the performance of consensus and relaxes the requirements of periodic execution. Following [12], some publications also show that ETMs are suitable for a class of first-order MASs [13], for double-integrator MASs [14], and for a class of linear time-invariant MASs [15]. By ETM, the above literature focuses on reducing updates of the controllers in agents but still requires continuous inter-agent exchange. Aiming at alleviating the burden of network channels, Ref. [16] presents an event-triggered algorithm to get rid of the continuous measurements of neighbors’ states. Additionally, Ref. [17] proposes two event-triggered condition functions: one for reducing control updates and the other for avoiding continuous communication among agents. Considering the second-order leader-following MASs with nonlinear dynamic behaviors, Refs. [18,19] propose distributed event-triggered sampling control approaches, where the agents only broadcast their discrete state values and the local controllers only update their outputs when the triggering conditions are satisfied. Ref. [20] uses an event-triggered framework that is free of continuous measurement of the triggered condition and gives sufficient conditions on the consensus of MASs with the linear agents. There is a common flaw in the aforementioned work. Centralized information depending on the spectrum of the Laplacian matrix is required when design an ETM control protocol. See Remark 2 in [17], Equation (11) in [19], and Theorem 2 in [20], to name but a few. This means that the ETMs and control protocols are based on the assumption that each agent knows the overall communication topology. Relaxing such a centralized assumption, therefore, is worthwhile to be investigated so as to take full advantage of the power of distributed protocols. Fortunately, in the studies of fully distributed consensus protocols in MASs, there has been much progress [21,22,23,24,25]. Depending on only local information of each agent, some distributed adaptive consensus protocols are presented and applied to undirected communication graphs [21,22,26] and directed communication graphs [23,25,27].

However, these protocols require continuously local states and interacting states, which does not suit the case in which resources of communication and computation are limited. In particular, when combining with ETMs, it is a promising topic to obtain less conservation in terms of reducing the frequency of inter-agent exchanges.

Recently, the problem of the distributed ETM consensus protocols has aroused the increasing interest of researchers. Ref. [28] presents an ETM protocol with unknown and nonidentical control directions of nonlinear MASs. Ref. [29] considers the event-triggered fault-tolerant consensus to improve the robustness of the consensus of MASs with general linear dynamics. To cope with time-varying layer-to-layer delays among hierarchical leader-following MASs, Ref. [30] proposes a distributed ETM protocol to achieve the consensus of networks. Ref. [31] presents an ETM consensus protocol to guarantee that network executions converge to the average of the initial agents’ states exponentially fast with switching communication topologies.

Motivated by the existing literature, in this paper, the ETM consensus protocol that is free of centralized information depending on the spectra of the Laplacian matrix is investigated. Moreover, agents in the considered MAS are with nonlinear dynamics under the connected undirected graph. The primary contributions are summarized as follows:

(a) To overcome the challenging problem that information depending on the spectra of Laplacian matrices is required prior to selecting parameters of event-triggered functions, a novel event-triggered sampled-data mechanism with an adaptive threshold is first proposed.
(b) A fully distributed consensus protocol for second-order MASs with nonlinear dynamics is designed, which is based on event-triggered sampled data interacting information among agents.
(c) Only the relative discrete position information is employed in both the event-triggered rule and the consensus protocol, which results in that the undesired velocity measurements can be avoided.

Throughout this paper, $R^{n}$ and $R^{n\times n}$ denote the *n*-Euclidean space and the set of all n×n real matrices, respectively; ∥·∥ stands for either the Euclidean vector norm or the spectral norm of a matrix; ⊗ denotes the Kronecker product; $I_{n}$ represents an n×n identity matrix; $\lambda_{min}\left(\cdot \right)$ and $\lambda_{max}$ denote the minimum and maximum eigenvalue of a matrix, respectively; and $diag\{d_{1},\ldots ,d_{n}\}$ denotes the diagonal matrix with the elements $d_{1},\ldots ,d_{n}$ on the diagonal.

## 2. Preliminaries

The following lemmas are necessary for the analysis of this paper.

### 2.1. Supporting Lemmas

**Lemma** **1.** *Let ω:R→R be a uniformly continuous function on [0,∞}. Suppose that*

$$lim_{t\to \infty }\int_{0}^{t}\omega \left(\tau \right)d\tau$$

*exists and is finite. Then,*

ω(t)→0ast→∞.

**Lemma** **2.** *The following linear symmetric matrix inequality (LMI)*

$$S=S^{T}=\left(\begin{array}{cc} A & B\\ {}* & C \end{array}\right)<0,$$

*is equivalent to one of the following conditions:*

1. *S<0;*
2. *$A<0,C-B^{T}A^{-1}B<0$;*
3. *$C<0,A-BC^{-1}B^{T}<0$.*

**Lemma** **3** ([32]). *For the function V(x,t), V(x,t)→0 as t→∞ holds, when the following conditions are met:*

1. *V(x,t) has a lower bound;*
2. *$\dot{V}\left(x,t\right)$ is negative semi-definite;*
3. *$\dot{V}\left(x,t\right)$ is a uniformly continuous function with regard to time, in other words, $\ddot{V}\left(x,t\right)$ has a bound.*

**Lemma** **4.** *The Laplacian matrix L of an undirected graph G is semi-positive definite, which has a simple zero eigenvalue, and all of the other eigenvalues are positive if and only if the undirected graph G is connected.*

**Lemma** **5** ([33]). *If L is reduciable, $L_{ij}=L_{ji}\leq 0$ for i≠j, and $\sum_{j=1}^{N}L_{ij}=0,i=1,2,\ldots ,N$ then, for any constant ϖ>0, all eigenvalues of the matrix H=L+B are positive, i.e., λ(H)>0, where B=diag{ϖ,0,…,0}.*

### 2.2. Graph Theory

The notations of communication graphs in this paper are extensively used in literature. The networking topology among *N* followers is modeled by a positively weighted undirected graph G=(V,E,W), where V denotes a nonempty vertex set $\left\{v_{1},v_{2},\ldots ,v_{N}\right\}$ describing agents; E⊂V×V denotes the set of undirected edges $e_{ij}$ describing the information exchanging and $W={\left(w_{ij}\right)}_{N\times N}$ denotes the underlying weighted adjacency matrix with nonnegative elements. An undirected edge $e_{ij}$ in graph G means that nodes $v_{i}$ and $v_{j}$ can exchange information with each other. If $e_{ij}$ exists between two nodes, $w_{ij}=w_{ji}>0$; otherwise, $w_{ij}=w_{ji}=0$. A graph is connected if every vertex in V is globally reachable and a vertex i∈V is globally reachable if any vertex other than *i* has at least one path starting at the vertex and ending at the vertex *i*. Furthermore, we assume that $i\notin N_{i}$ (no self-loop is contained), and hence for all i∈V, $w_{ii}=0$. The Laplacian matrix $L=[l_{ij}]$ is defined by:

$$l_{ij}=\left\{\begin{array}{cc} \; & \sum_{j\in N_{i}}w_{ij}\;\;i=j,\\ \; & -w_{ij}\;\;\;i\neq j. \end{array}\right.$$

For the networking topology with a leader, the total communication topology between the leader and its followers can be formulated by graphs $\bar{G}$, namely, $G\subset \bar{G}$. In $\bar{G}$, one leader can only send information to out-neighboring followers but not receive it reversely. Let $K={\left[k_{1},\ldots ,k_{N}\right]}^{T}$ denote the set of the weights from the leader to its followers. Accordingly, the Laplacian matrix of $\bar{G}$ is defined by:

$$\bar{L}=\left(\begin{array}{cc} 0 & 0\\ -K & H \end{array}\right),$$

where $H={\left\{h_{ij}\right\}}_{N\times N}=L+D$ and $D=diag\{k_{1},\ldots ,k_{N}\}$.

### 2.3. Problem Formulation

The second-order MAS considered in this paper consists of one leader and *N* followers, which can be formulated by:

(1) $$\begin{array}{cc} {\dot{x}}_{i}\left(t\right)= & v_{i}\left(t\right),\\ {\dot{v}}_{i}\left(t\right)= & f\left(t,x_{i}\left(t\right),v_{i}\left(t\right)\right)+u_{i}\left(t\right), \end{array}$$

where $x_{i}\left(t\right)$,$v_{i}\left(t\right),u_{i}\left(t\right)\in R^{n}$ denote the position, velocity and control input of the agent *i*, respectively, and $f\left(\cdot \right)$ is a continuously differentiable vector-valued nonlinear function to describe the self-dynamics of agents. The dynamics of the leader is governed by:

(2) $$\begin{array}{cc} {\dot{x}}_{0}\left(t\right)= & v_{0}\left(t\right),\\ {\dot{v}}_{0}\left(t\right)= & f\left(t,x_{0}\left(t\right),v_{0}\left(t\right)\right), \end{array}$$

where $x_{0}$, $v_{0}$ are the position and velocity of the leader, respectively. Throughout this paper, the following assumption is made.

**Assumption** **1.** *For the nonlinear function $f(t,x_{i}\left(t\right),v_{i}\left(t\right))$, the velocity state $v_{i}\left(t\right)$ is linearly coupled, which means*

$$f\left(t,x_{i}\left(t\right),v_{i}\left(t\right)\right)\leq \varsigma v_{i}\left(t\right)+f\left(t,x_{i}\left(t\right)\right)\;\;\;\forall x_{i},v_{i}\in R^{n},$$

*where ς is a scalar or a matrix with proper dimensions. Additionally, for any $x,y,z,v\in R^{n}$, there exists a nonnegative constant ρ such that*

$$∥f\left(t,x_{i}\right)-f\left(t,x_{j}\right)∥\leq \rho ∥x_{i}-x_{j}∥.$$

In the existing literature, the event-triggered controller for the agent *i* is usually designed as (taking [19] as an example):

(3) $$\begin{array}{cc} u_{i}\left(t\right)= & -\tilde{\alpha }\sum_{j=1}^{N}w_{ij}\left[x_{j}\left(t_{k}^{j}\right)-x_{i}\left(t_{k}^{i}\right)+v_{j}\left(t_{k}^{j}\right)-v_{i}\left(t_{k}^{i}\right)\right]\\ \; & -\tilde{\alpha }k_{i}\left[x_{i}\left(t\right)-x_{0}\left(t\right)+v_{i}\left(t\right)-v_{0}\left(t\right)\right],t\in \left[t_{k}^{i},t_{k+1}^{i}\right), \end{array}$$

where $\tilde{\alpha }>0$ is coupling strength and $t_{k}^{j}≜\arg\;\min_{p}\left\{t-t_{p}^{j}|t\geq t_{p}^{j},p\in N\right\}$, i.e., $t_{k}^{j}$ is the latest triggering time of agent j before time *t*. The control protocol is distributed since each agent only uses local information of neighboring agents, which can be clearly seen in (3). Similar distributed protocols can be found in [4,17,18,20]. In these papers, the feasibilities of the consensus criteria depend on that the coupling gains and the eigenvalue of a special matrix associated with the Laplacian matrix must satisfy some additional conditions. For example, in [19], $\lambda_{min}\left(L+D+{\left(L+D\right)}^{T}\right)>\frac{2\rho }{\tilde{\alpha }}$, where L denotes a Laplacian matrix and D denotes the leader adjacency matrix. To satisfy the condition, the information of the Laplacian matrix and leader adjacency matrix has to be known a priori for coupling gains design. One may question why not apply a sufficiently small value $\frac{2\rho }{\tilde{\alpha }}$, without using the global spectra information for solving this problem. It is noticed that a sufficiently small value $\frac{2\rho }{\tilde{\alpha }}$ means a large value of $\tilde{\alpha }$, which will directly increase the energy cost of the control. Hence, it is energy-efficient and of great significance to design a fully distributed approach without using the Laplacian matrix and the leader adjacent matrix. In this paper, we design an event-triggered communication mechanism to achieve leader-following consensus for second-order MASs and a consensus control protocol with updated coupling gains.

**Definition** **1.** *Consensus of a leader-following second-order MAS is said to be asymptotically achieved if both $\lim_{t\to \infty }∥{\hat{x}}_{i}-{\hat{x}}_{0}∥=0$ and $\lim_{t\to \infty }∥{\hat{v}}_{i}-{\hat{v}}_{0}∥=0,i\in N$ are satisfied for any initial values.*

## 3. Main Results

**Figure 1:**
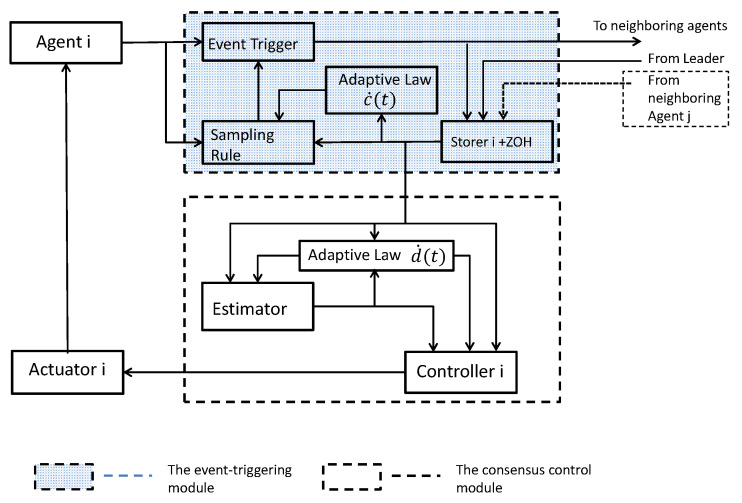
A fully distributed event-triggered transmission strategy for agent-*i*.

In this section, the main results of this paper are proposed. Generally speaking, the event-triggered transmission strategy consists of two modules [19]: (a) the consensus control protocol and (b) the event-triggered rule. For a better understanding, the overall framework of the proposed event-triggered transmission strategy is illustrated in Figure 1, which will be specifically explained in the following subsections.

### 3.1. The Event-Triggered Module

The sampling process of event-trigger mechanisms relies on the event-triggered condition rather than the elapse of a fixed time. Thus the *k*-th sampled-data indicates the data sampled at the *k*-th triggered event. Denote the *k*-th event-triggered instant of agent *i* with $t_{k}^{i}$. There exist measurement errors of the event-triggered sampling states $x_{i}\left(t_{k}^{i}\right),v_{i}\left(t_{k}^{i}\right)$ to its current states $x_{i}\left(t\right),v_{i}\left(t\right)$, which can be defined by $e_{xi}\left(t\right)=x_{i}\left(t_{k}^{i}\right)-x_{i}\left(t\right)$, and $e_{vi}\left(t\right)=v_{i}\left(t_{k}^{i}\right)-v_{i}\left(t\right),\;\;i=1,2,\ldots ,N,$ where $t\in [t_{k}^{i},t_{k+1}^{i})$. The next broadcasting instant of agent *i* is determined by:

(4) $$t_{k+1}^{i}=inf\left\{t>t_{k}^{i}:E_{i}\left(t\right)\geq 0\right\},$$

where

(5) $$\begin{array}{cc} \; & E_{i}\left(t\right)={∥e_{xi}\left(t\right)∥}^{2}-\underset{\Upsilon (t)}{\underset{︸}{d_{i}\left(t\right)sign\left(d_{i}\right){∥\sum_{j=1}^{N}L_{ij}x_{j}\left(t_{k}^{j}\right)+k_{i}\left(x_{i}\left(t_{k}^{i}\right)-x_{0}\left(t\right)\right)∥}^{2}}}, \end{array}$$

and $d_{i}\left(t\right)$ is an updated threshold to be designed and $t_{k}^{j}≜\arg\;\min_{p}\left\{t-t_{p}^{j}|t\geq t_{p}^{j},p\in N\right\}$, i.e., $t_{k}^{j}$ is the latest triggering time of agent j before time *t*. From (5), it can be seen that only relative position information is employed. The workflow of the event-triggered module can be described as follows:

(1) The storer *i* receives the latest state values from the neighboring agents and the leader (if agent *i* is the leader’s neighbor). Basing on the information received, storer *i* generates the continuous output signals.
(2) The adaptive law ${\dot{d}}_{i}\left(t\right)$ updates the threshold $d_{i}\left(t\right)$ according to the information from the local storer.
(3) The sampling rule formulated by (4) processes the sampled data with regard to the event-triggered condition from the storer with a zero-order hold (ZOH).
(4) The event trigger obtains a triggering signal from the sampling rule and then performs sampling.

**Remark** **1.** *In the existing literature, there are two forms of control input in the event-triggered control protocol for MAS. One can be formulated by $u_{i}=\beta \sum_{j\in N_{j}}a_{i}\left[x_{j}\left(t_{k}^{i}\right)-x_{i}\left(t_{k}^{i}\right)\right]$; another is $u_{i}=\beta \sum_{j\in N_{j}}a_{i}\left[x_{j}\left(t_{k}^{j}\right)-x_{i}\left(t_{k}^{i}\right)\right]$, in which it can be seen that the main difference is the event-triggered sampled time of neighbors’ states. In the former scheme, the control input only updates the state signals (from the local agent and the neighboring agents) at the local sampling time instant $t_{k}^{i}$; in the latter scheme, these state values need to be updated whenever the local agent samples its state value or receives a new measurement state value from the neighboring agents. The two schemes have their advantages in different aspects: the latter scheme is superior in the aspect of reducing the burden of networking transmission and the former one serves the purpose of fewer controller updates. Hence, the latter scheme is adopted in this paper from the perspective of alleviating burdens on communication.*

**Remark** **2.** *In the case that agent i is not the leader’s neighbor, the storer i also accounts for zero-order holding of the latest discrete state values received from the neighbors as well as storing them. In the case that it is the leader’s neighbor, the store i adds the continuous state values from the leader and the latest discrete state values together and outputs the sum. It explains why the storer i generates the continuous signals.*

### 3.2. The Consensus Control Module

Now we are at the position to present the fully distributed consensus protocol of this paper as follows:

(6) $$\left\{\begin{array}{cc} {\dot{x}}_{i}\left(t\right)= & v_{i}\left(t\right),\\ {\dot{v}}_{i}\left(t\right)= & f\left(t,x_{i}\left(t\right),v_{i}\left(t\right)\right)-\alpha c_{i}\left(t\right)\sum_{j=1}^{N}L_{ij}x_{j}\left(t_{k}^{j}\right)-\alpha c_{i}\left(t\right)k_{i}\left[x_{i}\left(t_{k}^{i}\right)-x_{0}\left(t\right)\right]-\alpha w_{i}\left(t\right),\\ {\dot{w}}_{i}\left(t\right)= & -\gamma w_{i}-\beta c_{i}\left(t\right)\sum_{j=1}^{N}L_{ij}x_{j}\left(t_{k}^{j}\right)-\beta c_{i}\left(t\right)k_{i}\left[x_{i}\left(t_{k}^{i}\right)-x_{0}\left(t\right)\right], \end{array}\right.$$

where ${\dot{w}}_{i}$ is the estimator of the networking coupled velocities; α>0,β>0, γ>0 are positive coupling gains and $c_{i}\left(t\right)$ is time-varying parameters to be designed. With Figure 1, the protocol (6) can be specifically explained by the following workflow:

1. The adaptive law updates the time-varying gain $c_{i}\left(t\right)$ based on information from interaction and the local estimator;
2. The estimator calculates estimates the networking coupling velocities term $w_{i}\left(t\right)$;
3. The controller generates the control input and transmits it to the actuator *i*.

Let ${\tilde{x}}_{i}\left(t_{k}^{i},t\right)=x_{i}\left(t_{k}^{i}\right)-x_{0}\left(t\right)$, $f\left(t,{\tilde{x}}_{i}\left(t\right),{\tilde{v}}_{i}\left(t\right)\right)=f\left(t,x_{i}\left(t\right),v_{i}\left(t\right)\right)-f\left(t,x_{0}\left(t\right),v_{0}\left(t\right)\right)$. The error dynamical equations can be written as:

(7) $$\left\{\begin{array}{cc} \; & {\dot{\tilde{x}}}_{i}\left(t\right)=v_{i}\left(t\right),\\ \; & {\dot{\tilde{v}}}_{i}\left(t\right)=f\left(t,{\tilde{x}}_{i}\left(t\right),{\tilde{v}}_{i}\left(t\right)\right)-\alpha c_{i}\left(t\right)\sum_{j=1}^{N}h_{ij}{\tilde{x}}_{j}\left(t_{k}^{j},t\right)-\alpha w_{i}\left(t\right),\\ \; & {\dot{w}}_{i}\left(t\right)=-\gamma w_{i}-\beta c_{i}\left(t\right)\sum_{j=1}^{N}h_{ij}{\tilde{x}}_{j}\left(t_{k}^{j},t\right), \end{array}\right.$$

where $h_{ij}$ denotes the element of matrix *H*. From Lemma 5, *H* is positive definite if there is at least one informed agent. Throughout this paper, we make an assumption that there is at least one agent connected to the leader; otherwise, it is impossible to expect that the agents in the graph can follow the leader.

**Remark** **3.** *Since $\sum_{j=1}^{N}L_{ij}=0$, one can easily derive*

(8) $$\begin{array}{cc} \sum_{j=1}^{N}h_{ij}{\tilde{x}}_{j}\left(t_{k}^{i},t\right)= & \sum_{j=1}^{N}L_{ij}\left[x_{j}\left(t_{k}^{j}\right)-x_{0}\left(t\right)\right]+k_{i}\left[x_{i}\left(t_{k}^{i}\right)-x_{0}\left(t\right)\right]\\ = & -\sum_{j=1}^{N}w_{ij}\left[x_{j}\left(t_{k}^{j}\right)-x_{i}\left(t_{k}^{i}\right)\right]-k_{i}\left[x_{0}\left(t\right)-x_{i}\left(t_{k}^{i}\right)\right] \end{array}$$

To facilitate analysis, define a new error state vector $z\left(t\right)={\left[\tilde{x}\left(t\right),\tilde{v}\left(t\right),w\left(t\right)\right]}^{T}\in R^{3nN}$, where $\tilde{x}\left(t\right)=\left[{\tilde{x}}_{1},\ldots ,{\tilde{x}}_{N}\right]\in R^{nN}$, $\tilde{v}\left(t\right)=\left[{\tilde{v}}_{1},\ldots ,{\tilde{v}}_{N}\right]\in R^{nN}$, $w\left(t\right)=\left[w_{1},\ldots ,w_{N}\right]\in R^{nN}$. Then the protocol in (7) can be recast in the compact form

(9) $$\dot{z}\left(t\right)=\tilde{F}\left(t,\tilde{x}\left(t\right),\tilde{v}\left(t\right)\right)+\tilde{H}z\left(t\right)+\tilde{G}\varepsilon \left(t\right),$$

where $\varepsilon \left(t\right)={\left[e_{x1}-e_{x0},\ldots ,e_{xN}-e_{x0}\right]}^{T}\in R^{nN}$,

$$\tilde{H}=\left(\begin{array}{ccc} 0_{nN} & I_{nN} & 0_{nN}\\ -\alpha CH⊗I_{n} & 0_{nN} & -\alpha I_{nN}\\ -\beta CH⊗I_{n} & 0_{nN} & -\gamma I_{nN} \end{array}\right)\in R^{3nN\times 3nN},$$

$\tilde{G}={\left(\begin{array}{ccc} 0_{nN} & -\alpha CH⊗I_{n} & -\beta CH⊗I_{n} \end{array}\right)}^{T}$, $\tilde{F}\left(t,\tilde{x}\left(t\right),\tilde{v}\left(t\right)\right)={\left(\begin{array}{ccc} 0_{nN} & f\left(t,\tilde{x}\left(t\right),\tilde{v}\left(t\right)\right) & 0_{nN} \end{array}\right)}^{T}$, $f\left(t,\tilde{x}\left(t\right),\tilde{v}\left(t\right)\right)={\left[f\left(t,{\tilde{x}}_{1}\left(t\right),{\tilde{v}}_{1}\left(t\right)\right),\ldots ,f\left(t,{\tilde{x}}_{N}\left(t\right),{\tilde{v}}_{N}\left(t\right)\right)\right]}^{T}\in R^{nN}$, and *C* is the diagonal matrix $C=diag\left\{c_{1},\ldots ,c_{N}\right\}\in R^{nN\times nN}$.

### 3.3. Consensus Analysis

Based on the event-triggered rule (5) and the protocol (6), the following theorem gives the adaptive laws $\dot{c}\left(t\right)$ and $\dot{d}\left(t\right)$ to guarantee the consensus of the considered MAS in this paper.

**Theorem** **1.** *Consider a second-order leader-following multi-agent system (1) and (2) with the distributed sampling control protocol (6) and the event-triggered sampling rule (5). Suppose that the graph G is connected and Assumption 1 holds. Then the second-order consensus can be reached under the following distributed adaptive laws:*

(10) $$\begin{array}{c} {\dot{c}}_{i}\left(t\right)=\zeta_{i}{\tilde{x}}_{i}^{T}\left(\beta -\alpha \right)\sum_{j=1}^{N}h_{ij}{\tilde{x}}_{j}\left(t_{k}^{j},t\right)+\zeta_{i}w_{i}^{T}\left(t\right)\delta \frac{\beta^{2}-\alpha^{2}}{\beta }\sum_{j=1}^{N}h_{ij}{\tilde{x}}_{j}\left(t_{k}^{j},t\right), \end{array}$$

(11) $$\begin{array}{c} {\dot{d}}_{i}\left(t\right)=-\xi_{i}\sum_{j=1}^{N}h_{ij}{\tilde{x}}_{j}^{T}\left(t_{k}^{j},t\right)\sum_{j=1}^{N}h_{ij}{\tilde{x}}_{j}\left(t_{k}^{j},t\right). \end{array}$$

*where δ, $\zeta_{i}$, and $\xi_{i}$ are positive constants.*

**Proof.**  Consider the following Lyapunov function candidate:

(12) $$V=\frac{1}{2}z^{T}\left(t\right)\Omega z\left(t\right)+\sum_{i=1}^{N}\frac{\varpi }{2\zeta_{i}}{\left(c_{i}\left(t\right)-{\hat{c}}_{i}\right)}^{2}+\sum_{i=1}^{N}\frac{\omega }{2\xi_{i}}{\left(d_{i}\left(t\right)+{\hat{d}}_{i}\right)}^{2},$$

where $\Omega =\left(\begin{array}{ccc} \mu & -\varpi & \varpi \\ {}* & \eta & -\frac{\alpha }{\beta }\eta \\ {}* & * & \eta \end{array}\right)⊗I_{nN}$, ϖ, ω, ${\hat{c}}_{i}$, and ${\hat{d}}_{i}$ are positive constants to be determined. By letting the parameters in matrix Ω satisfy μ≫ϖ>0, η>0, it can be guaranteed that Ω>0. The positive semi-definiteness of *V* in (12) can also be easily ensured, which means V(z(t),ε,t)≥0 and V(z(t),ε,t)=0, if and only if z(t)=0 and all $c_{i}\left(t\right)={\hat{c}}_{i}$ and $d_{i}\left(t\right)={\hat{d}}_{i}$. For simplicity, we assume n=1 in the proof and that $I_{n}$ is equivalent to 1 such that it will be omitted hereafter.

Differentiating (12) along the trajectories of (9) yields

(13) $$\begin{array}{cc} \dot{V}\left(z\left(t\right),\varepsilon ,t\right)= & z^{T}\Omega \dot{z}\left(t\right)+\sum_{i=1}^{N}\frac{\varpi }{\zeta_{i}}\left(c_{i}\left(t\right)-{\hat{c}}_{i}\right){\dot{c}}_{i}\left(t\right)+\sum_{i=1}^{N}\frac{\omega }{\xi_{i}}\left(d_{i}\left(t\right)+{\hat{d}}_{i}\right){\dot{d}}_{i}\left(t\right)\\ = & z^{T}\left(t\right)\Omega \tilde{F}+z^{T}\left(t\right)\frac{1}{2}\left(\Omega \tilde{H}+{\tilde{H}}^{T}\Omega^{T}\right)z\left(t\right)+z^{T}\left(t\right)\Omega \tilde{G}\varepsilon \left(t\right)\\ \; & +\sum_{i=1}^{N}\frac{\varpi }{\zeta_{i}}\left(c_{i}\left(t\right)-{\hat{c}}_{i}\right){\dot{c}}_{i}\left(t\right)+\sum_{i=1}^{N}\frac{\omega }{\xi_{i}}\left(d_{i}\left(t\right)+{\hat{d}}_{i}\right){\dot{d}}_{i}\left(t\right), \end{array}$$

where

$$\begin{array}{cc} \; & \frac{1}{2}\left(\Omega \tilde{H}+\tilde{H}\Omega^{T}\right)=\\ \; & \left(\begin{array}{ccc} \varpi \left(\alpha -\beta \right)CH⊗I_{n} & \frac{1}{2}\mu ⊗I_{nN} & \frac{\varpi }{2}\left(\alpha -\gamma \right)⊗I_{nN}+\frac{\alpha^{2}-\beta^{2}}{2\beta }\eta CH⊗I_{n}\\ {}* & -\varpi ⊗I_{nN} & \left(-\alpha \eta +\frac{\varpi }{2}+\frac{\alpha \gamma }{2\beta }\eta \right)⊗I_{nN}\\ {}* & * & \left(\frac{\alpha^{2}}{\beta }\eta -\gamma \eta \right)⊗I_{nN} \end{array}\right), \end{array}$$

$$\Omega \tilde{G}={\left[\varpi \left(\alpha -\beta \right)CH⊗I_{n},0_{nN},\frac{\alpha^{2}-\beta^{2}}{\beta }\eta CH⊗I_{n}\right]}^{T},$$

and $\Omega \tilde{F}={\left[-\varpi f\left(t,\tilde{x}\left(t\right),\tilde{v}\left(t\right)\right),\eta f\left(t,\tilde{x}\left(t\right),\tilde{v}\left(t\right)\right),-\frac{\alpha }{\beta }\eta f\left(t,\tilde{x}\left(t\right),\tilde{v}\left(t\right)\right)\right]}^{T}$.

From (10), one obtains

(14) $$\sum_{i=1}^{N}\frac{\varpi }{\zeta_{i}}\left(c_{i}\left(t\right)-{\hat{c}}_{i}\right){\dot{c}}_{i}\left(t\right)=z^{T}\varpi \left(\begin{array}{c} \beta -\alpha \\ 0\\ \delta \frac{\beta^{2}-\alpha^{2}}{\beta } \end{array}\right)⊗\left(C-\hat{C}\right)H⊗I_{n}\left(\tilde{x}+\varepsilon \right)$$

where $\hat{C}=diag\left\{{\hat{c}}_{1},\ldots ,{\hat{c}}_{N}\right\}$.

Let $\frac{\eta }{\varpi }=\delta$. By substituting (14) into (13) and some simple calculation, one has

(15) $$\begin{array}{cc} \dot{V}\left(\tilde{z}\left(t\right),\varepsilon ,t\right)= & {\tilde{z}}^{T}\left(t\right)\Omega F+{\tilde{z}}^{T}\left(t\right)\frac{1}{2}\left(\Omega \bar{H}+\bar{H}\Omega^{T}\right)\tilde{z}\left(t\right)+{\tilde{z}}^{T}\left(t\right)\Omega \bar{G}\varepsilon \left(t\right)\\ \; & +\sum_{i=1}^{N}\frac{\omega }{\xi_{i}}\left(d_{i}\left(t\right)+{\hat{d}}_{i}\right){\dot{d}}_{i}\left(t\right), \end{array}$$

where

$$\begin{array}{cc} \; & \frac{1}{2}\left(\Omega \bar{H}+\bar{H}\Omega^{T}\right)=\\ \; & \left(\begin{array}{ccc} \varpi \left(\alpha -\beta \right)\hat{C}H⊗I_{n} & \frac{1}{2}\mu ⊗I_{nN} & \frac{\varpi }{2}\left(\alpha -\gamma \right)⊗I_{nN}+\frac{\alpha^{2}-\beta^{2}}{2\beta }\eta \hat{C}H⊗I_{n}\\ {}* & -\varpi ⊗I_{nN} & \left(-\alpha \eta +\frac{\varpi }{2}+\frac{\alpha \gamma }{2\beta }\eta \right)⊗I_{nN}\\ {}* & * & \left(\frac{\alpha^{2}}{\beta }\eta -\gamma \eta \right)⊗I_{nN} \end{array}\right), \end{array}$$

$\Omega \bar{G}={\left[\varpi \left(\alpha -\beta \right)\hat{C}H⊗I_{n},0_{nN},\frac{\alpha^{2}-\beta^{2}}{\beta }\eta \hat{C}H⊗I_{n}\right]}^{T}$. Following Assumption 1, one obtains

(16) $$\begin{array}{cc} z^{T}\left(t\right)\Omega F= & \sum_{i=1}^{N}\left(-\varpi {\tilde{x}}_{i}^{T}+\eta {\tilde{v}}_{i}^{T}-\frac{\alpha }{\beta }\omega_{i}^{T}\right)\left[f\left(t,x_{i}\left(t\right),v_{i}\left(t\right)\right)-f\left(t,x_{0}\left(t\right),v_{0}\left(t\right)\right)\right]\\ \leq & \varsigma \left(-\varpi {\tilde{x}}_{i}^{T}+\eta {\tilde{v}}_{i}^{T}-\frac{\alpha }{\beta }\omega_{i}^{T}\right){\tilde{v}}_{i}+\rho \left(\varpi ∥{\tilde{x}}_{i}{∥}^{2}+\eta ∥{\tilde{v}}_{i}{\tilde{x}}_{i}∥+\frac{\alpha }{\beta }∥\omega_{i}{\tilde{x}}_{i}∥\right)\\ \leq & \varsigma \left(-\varpi {\tilde{x}}_{i}^{T}+\eta {\tilde{v}}_{i}^{T}-\frac{\alpha }{\beta }\omega_{i}^{T}\right){\tilde{v}}_{i}+\kappa {∥z∥}^{2}, \end{array}$$

where $\kappa =\max\{\rho \left(\varpi +\frac{\eta }{2}+\frac{\alpha }{2\beta }\right),\frac{\eta }{2},\frac{\alpha }{2\beta }\}$.

Recasting the event-triggered condition (5) in the compact form, one obtains

(17) $$-\omega \varepsilon^{T}\left(t\right)\varepsilon \left(t\right)+\omega \bar{D}{\tilde{x}}^{T}\left(t_{k},t\right){\left(H⊗I_{n}\right)}^{2}\tilde{x}\left(t_{k},t\right)\geq 0,$$

where $\bar{D}=diag\left\{d_{1}sgn\left(d_{1}\right),\ldots ,d_{N}sgn\left(d_{N}\right)\right\}\in R^{nN\times nN}$.

Additionally, substituting the adaptive law (11) into $\sum_{i=1}^{N}\frac{\omega }{\xi_{i}}\left(d_{i}\left(t\right)+{\hat{d}}_{i}\right){\dot{d}}_{i}\left(t\right)$, one obtains

(18) $$\sum_{i=1}^{N}\frac{\omega }{\xi_{i}}\left(d_{i}\left(t\right)+{\hat{d}}_{i}\right){\dot{d}}_{i}\left(t\right)=-\omega {\left[\tilde{x}\left(t\right)+\varepsilon \left(t\right)\right]}^{T}\left(D+\hat{D}\right)⊗I_{n}\left[\tilde{x}\left(t\right)+\varepsilon \left(t\right)\right],$$

where $D=diag\left\{d_{1},\ldots ,d_{N}\right\},\hat{D}=diag\left\{{\hat{d}}_{1},\ldots ,{\hat{d}}_{N}\right\}$ Combining (17) and (18), then

(19) $$\begin{array}{cc} \sum_{i=1}^{N}\frac{\omega }{\xi_{i}}(d_{i}\left(t\right) & +{\hat{d}}_{i}){\dot{d}}_{i}\left(t\right)\leq \\ \; & -\omega \varepsilon^{T}\left(t\right)\varepsilon \left(t\right)-\omega {\left[\tilde{x}\left(t\right)+\varepsilon \left(t\right)\right]}^{T}\underset{\Delta_{D}}{\underset{︸}{\left(D-\bar{D}+\hat{D}\right)}}⊗I_{n}{\left(H⊗I_{n}\right)}^{2}\left[\tilde{x}\left(t\right)+\varepsilon \left(t\right)\right], \end{array}$$

holds. From the definitions of D and $\bar{D}$, $\Delta_{D}=\hat{D}$, if $d_{i}\left(t\right)\geq 0$; $\Delta_{D}=\hat{D}-2\bar{D}$, otherwise. In addition, recalling Definition (11), one can observe that ${\dot{d}}_{i}\left(t\right)\leq 0$, which means the value of $d_{i}\left(t\right)$ will never increase. Then $d_{i}\left(t\right)\leq d_{i}\left(t_{0}\right)$, where $d_{i}\left(t_{0}\right)$ denotes the value of $d_{i}\left(t\right)$ at initial time instant $t_{0}$. Here, by choosing the constants ${\hat{d}}_{i}\geq 2d_{i}\left(t_{0}\right)sgn\left(d_{i}\left(t_{0}\right)\right)\geq 2d_{i}sgn\left(d_{i}\right)$, it is not hard to derive $\Delta_{D}\geq 0,\forall d_{i}\left(t\right)\in \left(-\infty ,+\infty \right)$. Substituting (19) into (15), one obtains

(20) $$\begin{array}{cc} \dot{V}\left(z\left(t\right),\varepsilon ,t\right)= & z^{T}\left(t\right)\Omega F+z^{T}\left(t\right)\frac{1}{2}\left(\Omega \tilde{H}+\tilde{H}\Omega^{T}\right)z\left(t\right)+z^{T}\left(t\right)\Omega \bar{G}\varepsilon \left(t\right)-\omega \varepsilon^{T}\left(t\right)\varepsilon \left(t\right)\\ \; & -\omega {\left[\tilde{x}\left(t\right)+\varepsilon \left(t\right)\right]}^{T}\Delta_{D}{\left(H⊗I_{n}\right)}^{2}\left[\tilde{x}\left(t\right)+\varepsilon \left(t\right)\right]\\ \leq & \left(\begin{array}{cc} z^{T}\left(t\right) & \epsilon^{T} \end{array}\right)\Pi \left(\begin{array}{c} z(t)\\ \epsilon \end{array}\right), \end{array}$$

where

$$\Pi =\left(\begin{array}{cc} \Pi_{A} & \Pi_{B}\\ {}* & \Pi_{C} \end{array}\right)$$

$$\begin{array}{c} \begin{array}{cc} \Pi_{A11} & =\varpi \left(\alpha -\beta \right)\hat{C}H⊗I_{n}+\kappa I_{nN}-\omega \Delta_{D}{\left(H⊗I_{n}\right)}^{2}\\ \Pi_{A12} & =\Pi_{A21}=\left(\frac{1}{2}\mu -\frac{\varpi \varsigma }{2}\right)I_{nN}\\ \Pi_{A13} & =\Pi_{A31}=\frac{\varpi }{2}\left(\alpha -\gamma \right)⊗I_{nN}+\frac{\alpha^{2}-\beta^{2}}{2\beta }\eta \hat{C}H⊗I_{n}\\ \Pi_{A22} & =\left(-\varpi +\varsigma \eta +\kappa \right)I_{nN}\\ \Pi_{A23} & =\Pi_{A32}=\left(-\alpha \eta +\frac{\varpi }{2}+\frac{\alpha \gamma }{2\beta }\eta -\frac{\varsigma \alpha }{2\beta }\right)I_{nN}\\ \Pi_{A33} & =\left(\frac{\alpha^{2}}{\beta }\eta -\gamma \eta +\kappa \right)I_{nN}\\ \Pi_{B} & =\left(\begin{array}{c} \varpi \left(\alpha -\beta \right)\hat{C}H⊗I_{n}-\omega \Delta_{D}{\left(H⊗I_{n}\right)}^{2}\\ 0_{nN}\\ \frac{\alpha^{2}-\beta^{2}}{\beta }\eta \hat{C}H⊗I_{n} \end{array}\right)\\ \Pi_{C} & =-\omega (I_{nN}+\Delta_{D}{\left(H⊗I_{n}\right)}^{2}). \end{array} \end{array}$$

By properly selecting the parameters μ,ϖ, η, ω, ${\hat{c}}_{i}$, ${\hat{d}}_{i}$ in Lyapunov function candidate (12), it is not hard to derive that Π<0 holds with the help of Lemmas 4 and 5. It is observed that V(z(t),ε,t) and $\dot{V}\left(z\left(t\right),\varepsilon ,t\right)$ satisfy conditions (1) and (2) of Lemma 3, respectively. To verify condition (3) in Lemma 3, the following analysis is needed. From (20) and Π<0, one may easily derive that z,c(t),d(t) are all bounded. Additionally, from (19), the boundedness of z(t) is used to derive that ϵ(t) is bounded. Then from (9)–(11), one can derive the boundedness of $\dot{z}\left(t\right),\dot{c}\left(t\right),\dot{d}\left(t\right)$. Then, by invoking (13), it is finally obtained that $\ddot{V}\left(z\left(t\right),\varepsilon ,t\right)$ is bounded, i.e., condition (3) in Lemma 3 is satisfied. Hence the proof can be completed. □

**Remark** **4.** *One may question that the matrix H including the Laplacian matrix L as well as the matrix D from the whole graph information and topology needs to be known by each agent when solving LMIs to guarantee Π<0 and the method could not be considered as a fully distributed method. It should be pointed out that the parameters obtained by solving Π<0 are based on the fact that H>0. Namely, as long as the matrix H is positive definite, the proposed method guarantees the consensus of the network. It is well-known that H is positive definite if there is at least one informed agent, which is assumed throughout the paper. Therefore, the method is fully distributed.*

**Remark** **5.** *From Theorem 1, it can be seen that the event-triggered second-order consensus in the considered leader-following MAS can be reached under the distributed adaptive laws (10) and (11) without requiring any centralized conditions, as in some existing literature [17,18,19,20]. In the whole networking control design, including the event-triggered rule and the consensus protocol, only local information of neighboring agents is used.*

**Remark** **6.** *One may notice that the dimension of* Π* is 4N, which may result in that the selection of the parameters in the Lyapunov function candidate is not easy. In fact, the selection of parameters can transfer to the problem of solving feasible solutions of multiple linear matrix inequations. By solving these LMIs, one can easily obtain proper feasible solutions. Additionally, we provide an example of the feasible solutions for these parameters in the numerical result section.*

The following theorem shows the existence of a lower bound of inter-event times, which means that the Zeno behavior is excluded in Theorem 1.

**Theorem** **2.** *With the event-triggered consensus protocol and the conditions given in Theorem 1, there exists no agent in MAS (1) that exhibits Zeno behavior during the consensus process. That is, for each agent i∈V, the inter-event time $\Delta_{k}^{i}=t_{k+1}^{i}-t_{k}^{i}=\tau >0.$*

**Proof.**  Suppose the velocities of all agents in the network considered are bounded by $M_{v}>0$. At the triggering time instants ${\left\{t_{k}^{i}\right\}}_{k=0}^{\infty }$, $e_{xi}\left(t_{k}^{i}\right)=0,i=1,2,\ldots ,N$ from the definition of $e_{xi}\left(t\right)$. In each interval $t\in \left[t_{k}^{i},t_{k+1}^{i}\right)$, one obtains

(21) $$\begin{array}{cc} ∥e_{i}\left(t\right)∥= & ∥\int_{t_{k}^{i}}^{t}{\dot{e}}_{i}\left(s\right)ds∥\leq \int_{t_{k}^{i}}^{t}∥{\dot{e}}_{i}\left(s\right)∥ds\leq \int_{t_{k}^{i}}^{t}∥{\dot{x}}_{i}\left(s\right)∥ds\\ \leq & \int_{t_{k}^{i}}^{t}∥v_{i}\left(s\right)∥ds\leq M_{v}\left(t-t_{k}^{i}\right),\forall t\in \left[t_{k}^{i},t_{k+1}^{i}\right) \end{array}$$

According to event-triggered rule (4), the next event will not be triggered until the trigger function $E_{i}\left(t\right)=0$, which means that for agent *i* the next sampling time instant $t=t_{k+1}^{i}$ is at the moment when $∥e_{xi}\left(t_{k+1}^{i},t\right){∥}^{2}=\Upsilon_{i}\left(t\right)$ holds, where Υ(t) is defined in (5).

Assume that before consensus is reached, there exists a positive constant ${\underline{\Upsilon }}_{i}$ such that $\Upsilon_{i}\left(t\right)\geq {\underline{\Upsilon }}_{i}>0$, for some $t\in {\left\{t_{l}^{i}\right\}}_{l=0}^{\infty }$; otherwise, $\Upsilon_{i}\left(t_{k^{\prime }}^{i}\right)=0$ for some $t\in {\left\{t_{k^{\prime }}^{i}\right\}}_{k^{\prime }=0}^{\infty }$. At the event time $t_{k^{\prime }}^{i}$, the consensus has been achieved and there is no need to trigger the event. That is to say, before the consensus is achieved, from (21), one obtains

(22) $$\begin{array}{cc} {\left[M_{v}\left(t_{k+1}^{i}-t_{k}^{i}\right)\right]}^{2} & \geq ∥e_{xi}\left(t_{k+1}^{i}\right){∥}^{2}=\Upsilon_{i}\left(t\right)\geq {\underline{\Upsilon }}_{i}>0. \end{array}$$

Now we will prove $\lim_{k\to \infty }t_{k}^{i}=\infty$ by contradiction. Assuming $\lim_{k\to \infty }t_{k}^{i}=T^{i}<\infty$, one can easily derive ${\underline{\Upsilon }}_{i}\leq {\left[M_{v}\left(t_{k+1}^{i}-t_{k}^{i}\right)\right]}^{2}$, which implies ${\underline{\Upsilon }}_{i}\leq 0$. This contradicts (22). Consequently, $\lim_{k\to \infty }t_{k}^{i}=\infty$ is proven. Assuming $\Delta_{k}^{i}\to 0$ and invoking (22), one can verify that ${\underline{\Upsilon }}_{i}\leq 0$, which contradicts the condition ${\underline{\Upsilon }}_{i}>0$. $\Delta_{k}^{i}=t_{k+1}^{i}-t_{k}^{i}=\tau >0$ therefore holds. This completes the proof of Theorem 2. □

## 4. Numerical Results

In this section, a numerical example is presented to illustrate the feasibility and effectiveness of the proposed mechanism. We consider a multi-agent system with one leader and six agents. To verify that it is fully distributed without requiring the spectra of Laplacian matrices, we use the following two graphs, whose eigenmatrices are $G_{1}$ and $G_{2}$, which are given by

$$G_{1}=\left(\begin{array}{cccccc} 6 & -1 & -2 & -1 & -2 & 0\\ -1 & 8 & -3 & 0 & 0 & -4\\ -2 & -3 & 5 & 0 & 0 & 0\\ -1 & 0 & 0 & 4 & -3 & 0\\ -2 & 0 & 0 & -3 & 6 & -1\\ 0 & -4 & 0 & 0 & -1 & 5 \end{array}\right),G_{2}=\left(\begin{array}{cccccc} 2 & -1 & -1 & 0 & 0 & 0\\ -1 & 5 & -3 & -1 & 0 & 0\\ -1 & -3 & 6 & -1 & -1 & 0\\ 0 & -1 & -1 & 3 & 0 & -1\\ 0 & 0 & -1 & 0 & 5 & -3\\ 0 & 0 & 0 & -1 & -3 & 4 \end{array}\right).$$

**Figure 2:**
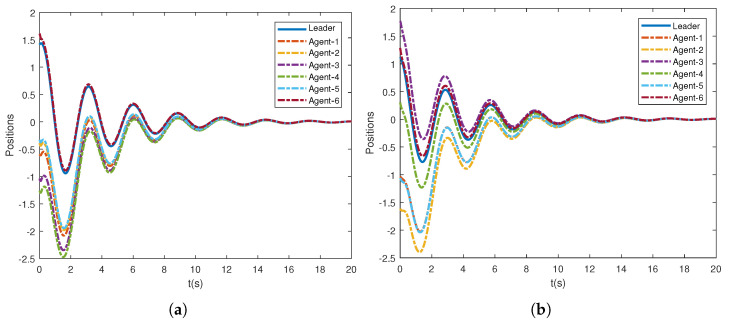
Consensus of positions under different topologies. (**a**) Positions of the leader and followers under $G_{1}$; (**b**) positions of the leader and followers under $G_{2}$.

**Figure 3:**
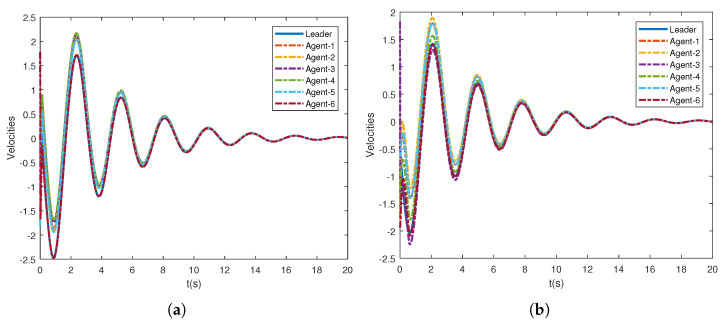
Consensus of velocities under different topologies. (**a**) Velocities of the leader and followers under $G_{1}$; (**b**) velocities of the leader and followers under $G_{2}$.

**Figure 4:**
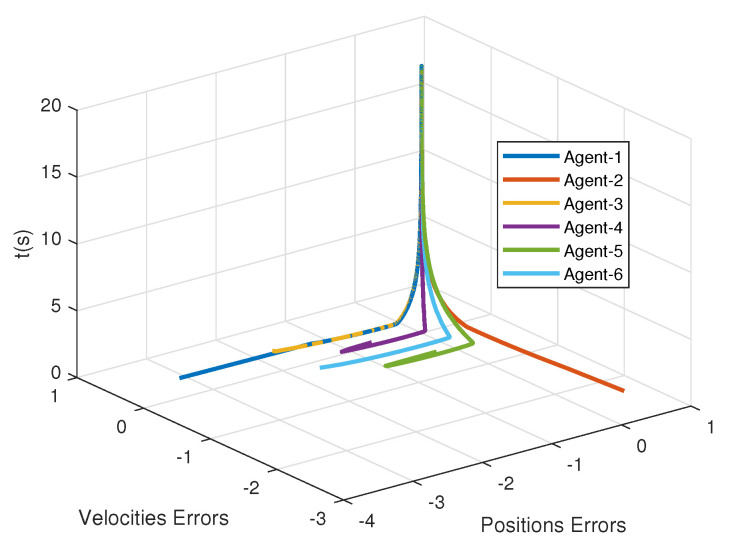
The second-order consensus under the proposed control protocol.

Accordingly, leader weight matrices are set to be diag{2,0,0,0,0} and diag{0,0,5,0,0}. The nonlinear dynamics of agents is the pendulum model that is given by $f\left(t,x_{i},v_{i}\right)=-\frac{g}{l}sin\left(x_{i}\right)-\frac{k}{m}v_{i}$, where g,k,l,m are the gravitational acceleration, the coefficient parameters, the length, and the mass of the rob, respectively. It is easy to verify that such a nonlinear dynamic model satisfies the above assumption. Here, we take g=9.8,k=0.1,l=4, m=1. To find a group of feasible parameters satisfying Π<0 in Theorem 1, one can use LMI toolbox in MATLAB. Here, we present a group of the parameters for the Lyapunov function candidate (12): $\mu =2,\varpi =1.5,\eta =20.5,\omega =40,\Delta_{D}=0.5I_{6},\varsigma =-0.5,\rho =-2$ and $\hat{C}=\frac{1}{20}H^{-1}$. For the parameters in consensus control protocol (6) and the adaptive law of the thresholds (11), in the simulation, their values are taken as α=1,β=30,$\gamma =35,\delta =13.67,\xi =\left[\xi_{1},\ldots ,\xi_{6}\right]=\left[0.5,0.2,0.4,0.3,0.5,0.6\right]$. In addition, the initial positions and velocities of the leader and followers are randomly generated between $\left[-1,1\right]$. From Figure 2, it can be observed that all the follower agents can track the position of the leader under both graph $G_{1}$ and graph $G_{2}$ without retuning the parameters. Additionally, Figure 3 shows that the tracking performance of velocities is also guaranteed. In Figure 4, the tracking errors of positions and velocities of six agents with graph $G_{1}$ are presented, and it demonstrates the second-order consensus performance of the proposed method. It can be seen from Figure 2 and Figure 3 that the consensus can be achieved eventually under the proposed event-triggered protocol, and the system achieved a consensus around 12 s.

**Figure 5:**
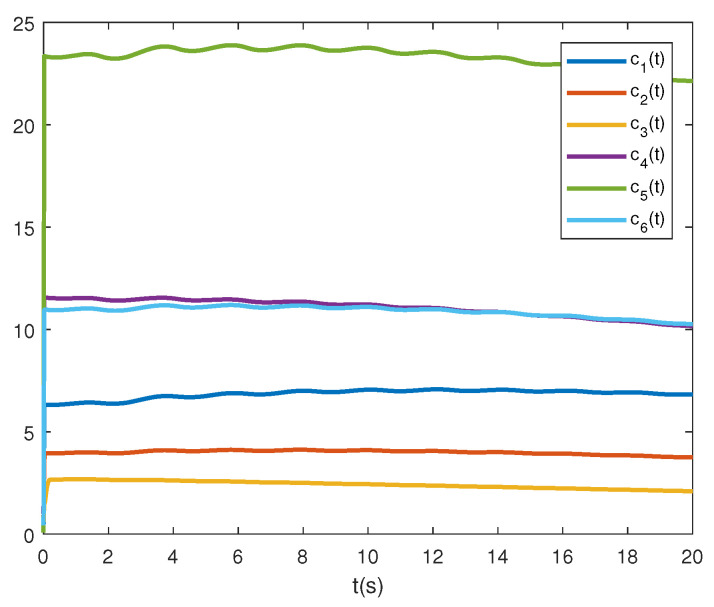
Consensus protocol coupling gains $c_{i}\left(t\right)$ under $G_{1}$.

Under $G_{1}$, the states of adaptive protocol coupling gains are presented in Figure 5, where the distributed control gains $c_{i}$ adaptively converge to proper ones. Figure 2 and Figure 3 demonstrate that the second-order leader-following consensus can be achieved with the network protocol proposed in this paper.

**Figure 6:**
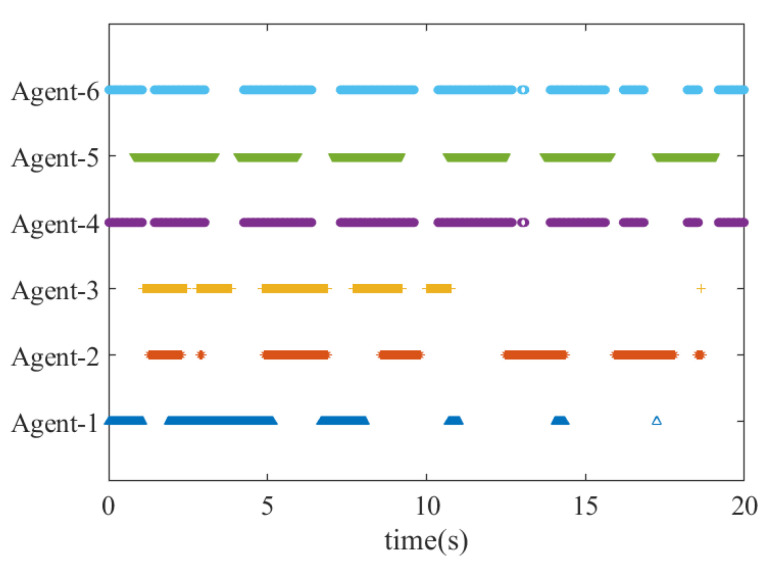
The state of triggered events of broadcasting signals under $G_{1}$.

To show the effectiveness of ETM in reducing the frequency of inter-agent exchanges, Figure 6 presents the states of the events in which each agent broadcasts its state to others under the topology graph $G_{1}$, where the blue areas represent that the predefined events are triggered.

**Figure 7:**
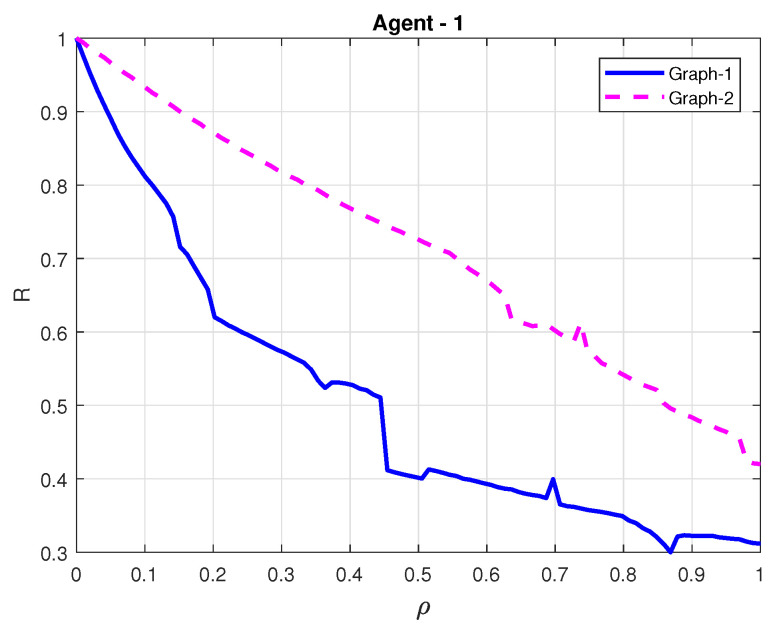
Ratio of broadcasting interacting signal and event-triggered criterion ρ by the ETM in Ref. [19].

**Figure 8:**
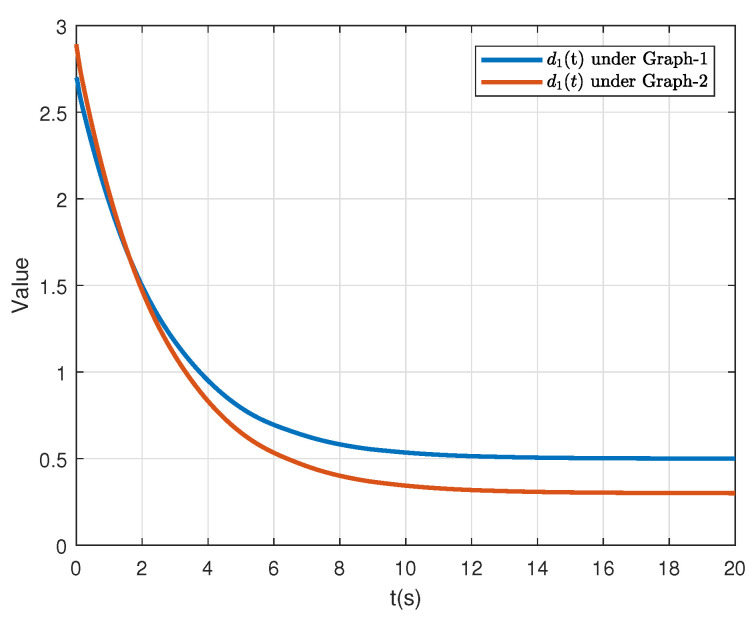
The time-varying threshold $d_{1}\left(t\right)$ of agent-1 under $G_{1}$ and $G_{1}$.

For comparison, we also conducted the simulation for the ETM with the constant event-triggered thresholds in [19] (see Equation (7)). In this work, the number of broadcasting interacting signals of each follower are negative related to the event-triggered thresholds parameters. By taking agent-1 as an example, the relationship between ϱ and the number of the triggered events *R* is presented in Figure 7 under $G_{1}$ and $G_{2}$, respectively. Note that to facilitate analysis, we use a new defined parameter $\varrho \in \left[0,1\right]$ to replace $\varrho_{1},\varrho_{2}$ where $\varrho_{1}=0.12\varrho ,\varrho_{2}=0.18\varrho$ in this simulation. Additionally, the adaptive thresholds $d_{1}\left(t\right)$ of agent-1 under $G_{1}$ and $G_{2}$ are accordingly given in Figure 8. The comparison of the ETM proposed in this paper and its counterpart in [19] demonstrates that the adaptive triggering thresholds are free of using the spectra of Laplacian matrices, which verifies the lower conservativeness of the proposed control protocol.

**Remark** **7.** *Under the proposed protocol, the whole network can reach a consensus using only the agent dynamics and the relative states of neighboring agents with event-triggered communication. However, from the protocol (6), it can be seen that the coupling gain with the form of multiplying a new nonlinear function $c_{i}\left(t\right)$ defined in (10) will increase rapidly when the initial consensus error is large. It may bring considerable control input and could be limited in the real application. Another potential limitation of the adaptive consensus protocol is scalability. When facing large-scale MASs, the protocol needs the whole system agent to re-adjusts when new agents join the system even though the original system has reached consensus. These limitations pose challenges for the practical application.*

## 5. Conclusions

This paper proposed a novel event-triggered control protocol for leader-following consensus of second-order MASs under undirected communication topologies. To address the problem of using continuous communicating signals among the follower agents, we have proposed a distributed consensus protocol with an event-triggered communicating mechanism. To get rid of the dependence on centralized information, we proposed adaptive laws to update the coupling gains and event-triggered thresholds online. Moreover, due to the noise-prone process of velocity measurement, only relative positions among agents are employed in the proposed protocol. Compared with some existing results, the protocol in this paper has reduced the times of inter-agent communication while reaching a consensus. Moreover, the ETM with adaptive thresholds possessed less conservativeness without using centralized information. By the numerical examples, it has been verified that, with the proposed protocol, a consensus can be achieved under the updated coupling gains and the distributed thresholds.
